# Shoot stem cell specification in roots by the WUSCHEL transcription factor

**DOI:** 10.1371/journal.pone.0176093

**Published:** 2017-04-26

**Authors:** Boaz Negin, Or Shemer, Yonatan Sorek, Leor Eshed Williams

**Affiliations:** The Robert H. Smith Institute of Plant Sciences & Genetics in Agriculture, Faculty of Agriculture, Food and Environment, The Hebrew University of Jerusalem, Rehovot, Israel; Instituto de Biologia Molecular y Celular de Plantas, SPAIN

## Abstract

The WUSCHEL homeobox transcription factor is required to specify stem-cell identity at the shoot apical meristem and its ectopic expression is sufficient to induce *de novo* shoot meristem formation. Yet, the manner by which WUS promotes stem-cell fate is not yet fully understood. In the present research we address this question by inducing WUS function outside of its domain. We show that activation of WUS function in the root inhibits the responses to exogenous auxin and suppresses the initiation and growth of lateral roots. Using time lapse movies to follow the cell-cycle marker CYCB1;1::GFP, we also show that activation of WUS function suppresses cell division and cell elongation. In addition, activation of WUS represses the auxin-induced expression of the *PLETHORA1* root identity gene and promotes shoot fate. Shoot apical meristem formation requires a high cytokinin-to-auxin ratio. Our findings provide evidence for the manner by which WUS specifies stem-cell identity: by affecting auxin responses, by reducing the cell mitotic activity and by repressing other developmental pathways. At the meristem, the stem-cells which are characterized by low division rate are surrounded by the highly proliferative meristematic cells. Our results also provide a model for WUS establishing the differential mitotic rates between two cell populations at the minute structure of the meristem.

## Introduction

The *WUSCHEL* gene (*WUS*) encodes a homeodomain transcription factor that is required to specify stem-cell identity at the shoot apical meristem (SAM). *WUS* expression initiates at the 16-cell globular stage embryo and is essential for meristem formation and maintenance [1]. Consequently, *wus* mutants fail to organize a functional SAM [2], up-regulation of *WUS* in the meristem leads to increase in stem cell population size [3–5], and ectopic *WUS* expression is sufficient to induce ectopic specification of stem cell fate and *de novo* meristem formation [6–8]. *WUS* is expressed at the SAM in a small group of cells that define the organizing center (OC) located underneath the stem cells [1,9], and migrates as a protein into the overlying cells to specify and maintain stem cell identity [10,11]. Within the stem cells WUS binds to the promoter of the *CLAVATA3* (*CLV3*) gene and activates its expression [11–13]. In turn, the *CLV3* which encodes for a peptide ligand, diffuses to the underlying cell layers and acts through the CLV1/CLV2 receptor complex to downregulate *WUS* transcription, thereby restricting the OC size [5,14–17]. This spatial feedback loop between the stem cells and the cells of the underlying OC, stabilizes the size of the stem cell population and the size of the OC and contribute to meristem homeostasis [18,19]. In recent years, many studies identified numerous elements affecting this homeostasis. For example, the plant hormone cytokinin (CK) activates *WUS* expression and WUS enhances cellular CK response in the OC [20]. However, a treatment with exogenously CK, do not increase SAM size and it was suggested by Uchida *et al* that the ERECTA receptor kinase family buffering the CK responsiveness in the SAM thereby regulating stem cells homeostasis [21].

Though WUS function is crucial for meristem maintenance and shoot development, the manner by which it specifies stem-cell fate is not yet fully understood. Recently, WUS putative direct targets were identified, suggesting that WUS represses the expression of genes associated with differentiation [22], and providing evidence for WUS potential to negatively affect auxin biosynthesis and perception [23].

Here we provide evidence for the manner by which WUS specifies shoot stem-cell identity in Arabidopsis: we report that induction of WUS function in the root suppresses cell division, inhibits the response to auxin and represses the expression of a root-identity gene, thereby allowing *de-novo* formation of shoot meristem.

## Results

**Fig 1:**
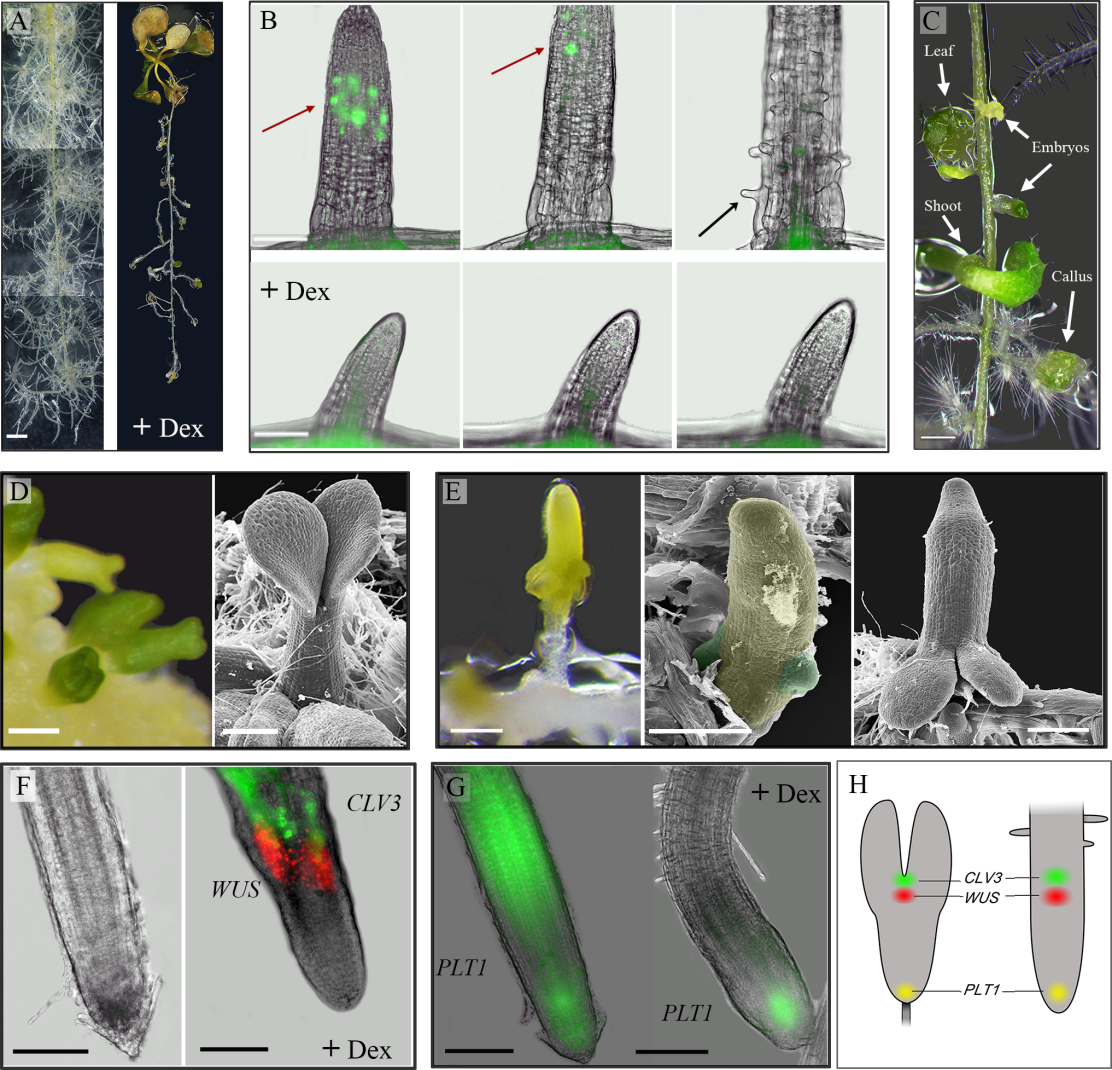
WUS inhibits Arabidopsis root responses to auxin, suppresses cell division and represses the expression of the *PLETHORA1* gene, thereby promotes shoot fate. *35S*::*WUS-GR Arabidopsis* seedlings (11- day old) were transferred to a medium supplemented with auxin (0.5μM NAA) in the presence or absence of 10μM Dex. **A**. Auxin induced massive lateral root formation (left). Activation of WUS by Dex led to inhibition of root formation and root growth (right). Images were taken after 13 days of culturing; separate images were merged. Bar = 2 mm. **B**. Induction of WUS by Dex suppresses cell division and elongation. Cell-division events in lateral roots were monitored by the cell-cycle marker CYCB1;1::GFP upon culturing on auxin in the presence or absence of 10μM Dex. Images were captured at 24h, 26h and 40 min, and 41h of incubation. Red arrow: dividing cells, black arrow: root hair. Bars = 100 μm (see also time-lapse S1 and S2 Movies). **C**. WUS in the presence of auxin induces the formation of callus, embryos and shoots. Image was taken after 12 days of culturing on auxin and Dex. Bar = 0.5 mm. **D.** Somatic embryos formed on wild-type callus exhibit typical basal-apical polarity: cotyledons distal and embryonic radicle proximal to the callus. Bar = 100 μm. **E**. WUS induces somatic embryo formation with atypical orientation. The embryonic radicle coincided with the lateral-root tip and the cotyledons developed shootward. Scanning electron microscopy views demonstrate the initiation of two cotyledon primordia on the lateral root (root and cotyledon primordia are false-colored yellow and green, respectively). Bars = 200 m. **F**. WUS activation induces the establishment of an organizing center and stem-cell population. The expression of *WUS* and *CLV3* was monitored by using the *35S*::*WUS-GR x WUS*::*DsRed x CLV3*::*GFP* marker line. No signal was detected in the presence of auxin after 12 days of culturing (left). Intense WUS (DsRed) and CLV3 (GFP) signals were detected in the presence of auxin and Dex after 12 days of culturing (right). Bars = 100 μm. **G**. WUS induction represses the *PLT1* expression. *35S*::*WUS-GR x PLT1*::*YFP* roots incubated for 104h on auxin without (left) or with (right) Dex. On the auxin-only medium, intense signals were detected in the root tip and elongation zone. In the presence of auxin and Dex, the signal was confined to the root tip. Bars = 100 μm. **H**. Scheme of *WUS* (red), *CLV3* (green) and *PLT1* (yellow) patterns of expression in a torpedo-stage zygote embryo (left) and upon WUS induction in a root cultured on auxin (+ Dex) (right).

In an attempt to elucidate how WUS acts to specify stem-cells fate, and to investigate its interaction with auxin signaling, we induced WUS function outside of its domain using the rat glucocorticoid receptor (GR)-inducible system [24]. We first tested how the GR ligand dexamethasone (Dex) affects root growth by transferring 11-day-old WT Col plants (n = 12) to medium supplemented with 10μM Dex and observed normal growth with no phenotypic effects (S1 Fig left panel). It is well established that auxin promotes lateral roots (LRs) initiation and LR primordium development [25]. Transferring 11-day-old *35S*::*WUS–GR* plants (n = 12) [7] to a medium with auxin (0.5μM NAA) resulted in the expected formation of numerous LRs in response to the auxin (Fig 1A). However, induction of WUS function by adding Dex, suppressed LRs initiation and growth (Fig 1A and S1 Fig), indicating that WUS suppresses the root response to exogenous auxin. This concurs with the report that WUS suppresses to a certain extent, the inhibition of hypocotyl elongation by auxin [6]. Transferring 11-day-old *35S*::*WUS–GR* plants to Dex medium without auxin (n = 12), also resulted in LRs growth inhibition (S1 Fig left panel).

To assess whether WUS suppresses LR growth by affecting cell division, we monitored the cell-cycle marker CYCB1;1::GFP [26] in LR primordia by making time-lapse movies in three replicates. We cultured the *35S*::*WUS–GR x CYCB1;1*::*GFP* plants on medium with auxin in the presence or absence of Dex for 24h. Time-lapse imaging was then performed for the next 24h at 5-min intervals (S1 and S2 Movies). With auxin, the LRs exhibited the typical cell divisions that occur at the root apical meristem (RAM) (Fig 1B) and the roots grew rapidly. After 7h, the meristem zone grew beyond the field of view and cells started to elongate and to differentiate into root hair cells (S1 Movie). However, in Dex-treated plants at imaging time zero (after 24h on Dex), the LRs were much shorter and only a low frequency of mitotic activity was observed, for all three replicates, indicating that WUS suppresses cell divisions. In addition, no cell elongation or differentiation was observed during the 24h of analysis. Thus, LR growth suppression was the result of both a reduction in cell-division rate and suppression of cell elongation. These results provide direct evidence that activation of WUS in the root suppresses cell division, suggesting that one mechanism through which WUS mediates stem-cell fate in the SAM is by reducing the cells mitotic activity.

Long-term culturing on auxin and Dex led to switches in developmental fate into three distinct pathways: callus formation, somatic embryogenesis and shoot organogenesis; all developed on the same root only at the primary or LR meristems (n = 12) (Fig 1C and S2 Fig). Without re-culturing, the effect of Dex and auxin may gradually decline, leading to the release of the cell division suppression and allowing the endogenous auxin to redirect cell fate. Therefore the variation in the local endogenous auxin concentration may lead to distinct fates. This is supported by the observation that when excised roots of *35S*::*WUS–GR* 11-day-old seedlings (n = 12), which do not have input from the endogenous shoot-derived auxin, were cultured on auxin and Dex, the LR formation was suppressed just like in the intact seedlings, but only few small calli were formed and there was no regeneration of shoots or somatic embryos on any of the LRs (S3 Fig).

The somatic embryo-like structures that developed on the intact seedlings LRs concur with previous reports [6,8]. Typically in tissue culture, somatic embryos develop with their shoot poles away from the source tissue (Fig 1D). However, in our experiments, the embryos appeared to be oriented with their shoot poles on the LR's proximal side toward the primary root (shootward), while their radicles coincided with the LR tips (Fig 1E). This variation in embryos orientation might result from the different experimental setting. For example, while Gallois et al [6] activated WUS function in a mosaic pattern, two days after germination on medium with 5 μM NAA, we activated WUS function in all cells of 11 day old seedlings with well-developed LR primordia simultaneously with exposure to 0.5 μM NAA. Scanning electron microscope analysis further confirmed our observation on the inverse orientation. Morphologically, the embryo structure was first recognized when two-to-four cotyledon primordia initiated on the LR/embryonic hypocotyl, followed by the emergence of leaf primordia between the cotyledons (Fig 1E and S4 Fig), indicating that a functional SAM producing leaf primordia had been established. To investigate the molecular events of SAM establishment, we analyzed the *DsRed–N7* reporter gene driven by the *WUS* promoter, a marker for the SAM organizing center (OC), and the GFP gene driven by the *CLAVATA3* (*CLV3)* promoter that serves as a marker for the SAM stem-cells [27]. After culturing the *35S*::*WUS–GR x WUS*::*DsRED–N7 x pCLV3*::*GFP–ER* plants on auxin for 12 days (n = 3), we could not detect any signal (Fig 1F, left panel). However, when Dex was added, a strong DsRed signal was detected above the root tip in all three replicates, before the somatic embryo could be identified morphologically, implying *de-novo* formation of an OC (Fig 1F, right panel). With respect to the GFP, we observed signals emanating from cells located above the DsRed signal (Fig 1F, right panel), consistent with the embryo's apical–basal orientation.

This confirmed that WUS activation had established an OC whose activity established an apical stem-cell population (expressing the *CLV3* marker) that further developed into active meristem exhibiting leaf primordia development (S4 Fig) oriented shootward.

The ubiquitously expression of the *WUS* gene in the *35S*::*WUS-GR* plant raises the question of why the embryonic SAMs are formed shootward?

The *PLETHORA1* (*PLT1*) is a root stem-cell maintenance regulatory gene [28] that was shown to be essential for LR primordia competency to regenerate shoot upon WUS induction [29]. We hypothesized that the root-identity genes that are expressed in the preexisting LR primordia, prevent WUS from acting in those cells and maintain a functional RAM that dictates the apical–basal embryo axis. To test this, we followed the expression of the *PLT1* gene upon WUS activation. It was recently shown that the expression domain of PLTs failed to expand rapidly when 4-day-old seedlings where cultured on 5μM indole acetic acid (IAA) combined with the auxin transport inhibitor, 1-Nnaphthylphthalamic acid (NPA). However after prolonged IAA plus NPA treatment (24–72 h), the expression of PLT–YFP shifted shootward [30]. Similar results were obtained showing that *PLT1*expression was elevated 24 hours after culturing on 50μM NAA [28]. When we transferred the *35S*::*WUS–GR x PLT1pro*:*PLT1*::*YFP* plant [31] to a medium with auxin (0.5μM NAA) (n = 5), after 72h we observed an intense YFP signals in the root-tip and at the elongation-zone, which remained throughout the analysis (104h). When we added Dex, the signal in the root-tip could still be seen, even at 104h (n = 5), but we did not detect a signal in the elongation zone (48–104h), (Fig 1G). This demonstrates that induction of WUS function in the root suppresses the auxin-induced *PLT1* expression in the elongation zone directly by affecting the *PLT1* transcription or by affecting auxin perception, or indirectly due to WUS positive feedback on CK signaling [32]. In addition, it demonstrates that WUS do not affect the expression of the *PLT1*root stem cell identity gene in its own expression domain.

## Discussion

In tissue culture low auxin to cytokinin ratio is required to promote shoot induction [33] wherein the activity of cytokinins is essential to promote *WUS* expression and other genes required for *de novo* meristem formation [34,35]. Although WUS is central for stem cell specification and despite the recent advances in identifying the WUS putative direct targets and interactors [22,23,36], the manner by which it acts is not yet fully understood.

In this study we show that activation of WUS in the root inhibits the responses to exogenous auxin and suppresses LRs formation. Recently, genes that inhibit auxin signaling and regulate polar auxin transport were identified as putative WUS direct targets [23], suggesting that WUS inhibits the auxin responses directly. But the observation that cytokine inhibits LR primordia initiation [37] offers an indirect inhibition, through activation of cytokine signaling mediated by WUS [23,32].

Activation of WUS function in the root in the presence of auxin resulted in regeneration of shoots and the formation of embryos and callus, all on the same root. However, when the *35S*::*WUS-GR* plants were cultured on MS supplemented with Dex without auxin, or when excised roots with no input from the endogenous shoot-derived auxin were cultured on DEX and auxin, no shoots or embryos were formed. This indicates that activation of WUS in the root is not sufficient to induce embryo/shoot meristems and for that a precise auxin inputs are required for shoot regeneration, a topic that requires further study.

The ubiquitous expression of *WUS–GR* (driven by the *CaMV 35* constitutive promoter) raises the question of why, in all of the examined roots (n = 12), only one embryo formed on each LR and always in the same orientation. Considering the complex interaction between auxin and cytokinin, which triggers root or shoot organogenesis [38] and the evident that during somatic embryogenesis, auxin and cytokinin regulate the apical–basal polarity pattern where SAM formation requires a high cytokinin-to-auxin ratio whereas the RAM formation requires an appropriate auxin gradient [39–41], we propose that *de-novo* embryonic SAM formation depends on the cells' competence to switch their identity. At the LR RAM, where the endogenous auxin level is high [42], the cells are not responsive to WUS and the fate remains of a root. But at the basal meristem-elongation zone boundary, where cytokinin synthesis takes place [40], WUS can repress the responses to the exogenous auxin and acts to establish the *de novo* OC (Fig 1H).

Using the *PLT1*marker we showed that exogenous auxin activates the *PLT1* expression in the root and that induction of WUS function represses this response, but do not affect the expression at the root tip. The *TOPLESS* (*TPL*) transcriptional co-repressor acts during embryogenesis to repress *PLT1* expression directly in the apical region of the embryo [43]. WUS has been shown to bind the *TPL* promoter directly and to activate its expression [23], and the two proteins have been shown to interact [44]. Therefore, WUS-mediated *PLT1* suppression potentially occurs via TPL.

Activation of WUS in the root suppresses cell division, suggesting that one mechanism through which WUS mediates stem-cell fate in the SAM is by reducing the cells mitotic activity. This is further supported by the fact that WUS-RELATED-HOMEOBOX5 (WOX5) reduces cell division at the root quiescent center by repressing the *CYCD3;3* expression directly [45], and that WUS can rescue the quiescent-center phenotype of the *wox5-1* mutant [46]. Similarly, WUS might restrain stem-cell division in the SAM by repressing *CYCD* expression, a hypothesis that requires further investigation.

**Fig 2:**
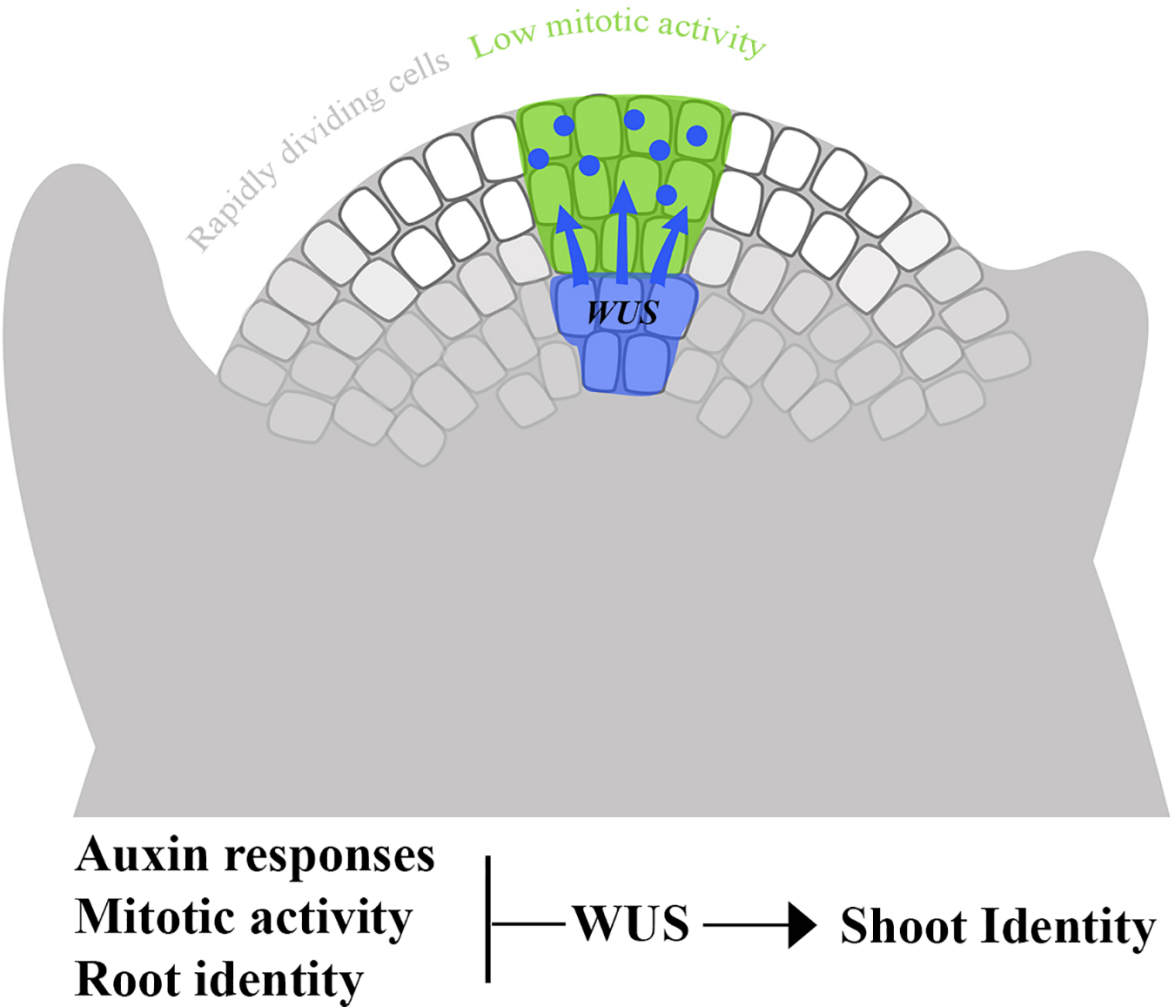
The WUS protein moves specifically to the overlaying cells and suppresses their mitotic activity. By moving specifically as functional protein to the group of cells at the tip of the meristem and suppressing their mitotic activity, WUS establishes differential mitotic rate between two domains, the stem cells and the neighboring meristematic cells.

At the SAM, the stem-cells which exhibit low mitotic activity are surrounded by the rapidly dividing cells of the peripheral zone [47]. The mechanism regulating this divergent mitotic activity in neighboring cell subpopulations is still not known. We propose that the WUS protein moves from its expression domain at the center of the meristem [10,11], while becoming functional, only to the overlying cells and not laterally. This results in the establishment of differential mitotic rate between the two domains (Fig 2). This is supported by the finding that misexpression of *WUS* in cells of the peripheral zone inhibited their proliferative ability [48].

In summary we demonstrated that WUS activation in the root suppresses cell division, inhibits the responses to auxin and represses the expression of root identity genes, suggesting that WUS mediates shoot stem-cell fate by reducing the cells mitotic activity, antagonizing auxin and repressing other developmental pathways. Although this study analyzed WUS function in the root, these mechanisms might be applied to the SAM. Further studies are needed to elucidate the downstream genes that potentially mediate these functions.

## Materials and methods

### Growth conditions and plant materials

Plant material used in this study were: Arabidopsis thaliana accession Colombia-0 (Col-0) and the following transgenic lines: *35S*::*WUS-GR* [7], *CYCB1;1::GFP [26]*, *PLT1*::*PLT1-YFP* [31], *WUS*::*dsRED X CLV3*::*GFP* [27] (CS23895, obtained from the Arabidopsis Biological Resource Center (ABRC). Seeds were surface sterilized and sown on 20 cm x 20 cm plates containing Murashige and Skoog medium (Duchefa M0256) and plates were placed at 4°c for 48 hours. For root analysis, plates were then placed vertically in long day conditions (18h light 6h dark) at 22°c. To generate plants having both the *35S*::*WUS*-*GR* and the different marker lines, homozygote plants were crossed and the analysis was done on the F1 plant. For validation genotyping by PCR was done on sampled F1 plants using specific primers for GR as follow: *WUS-GR*: F-TGAGTAGCCATGTCTATGGATC, R- TCATTTTTGATGAAACAGAAGC.

### Tissue culture

Eleven-day-old seedlings that were grown vertically were transferred to medium supplemented with auxin 0.093 mg/L NAA (Sigma–Aldrich N1641) and with Dexamethazone (Dex) (sigma-aldrich D2915) at concentrations of 10μM.

Somatic embryo formation from WT plant were generated as previously described [49].

### Imaging and microscopy

Plant images were captured using Olympus SZX7 Stereomicroscope equipped with DP73 camera. Fluorescent images and time lapses were captured using EVOS® FL Cell Imaging System equipped with CCD camera. Excitation wavelengths and emission filters were 488 nm/band-pass 505–530 nm for GFP or YFP and 552 nm/ band-pass 580–620 nm for dsRED. Scanning electron microscopy was performed as described previously [50] using a Hitachi 4700 scanning electron microscope.

## Supporting information

S1 Movie Mitotic activity in lateral roots. Eleven day old *35S*::*WUS-GR x CYCB1;1*::*GFP* seedlings were transferred to medium supplemented with auxin [0.5μM NAA] without Dex. After 24 hours of incubation, the mitotic activity was monitored for the next 24 hours by Live imaging videos that were captured using EVOS®FL Cell Imaging System equipped with CCD camera in 5 minutes intervals. Excitation wavelengths and emission filters were 488 nm/band-pass 505–530 nm.
(WMV)

S2 Movie Induction of WUS in Arabidopsis roots suppresses cell division and inhibits cell elongation and differentiation. Eleven day old *35S*::*WUS-GR x CYCB1;1*::*GFP* seedlings were transferred to medium supplemented with auxin [0.5μM NAA] and Dex. After 24 hours of incubation, the mitotic activity was monitored for the next 24 hours by Live imaging videos that were captured using EVOS®FL Cell Imaging System equipped with CCD camera in 5 minutes intervals. Excitation wavelengths and emission filters were 488 nm/band-pass 505–530 nm.
(WMV)

S1 Fig WUS inhibits the responses to auxin in Arabidopsis roots. Eleven day-old *35S*::*WUS-GR* seedlings were transferred to medium supplemented with auxin (0.5μM NAA) and 10μM DEX. Left panel: WT Col plants cultured on Dex without auxin show no phenotype; *35S*::*WUS-GR* plants cultured on Dex without auxin exhibit inhibition in root growth and initiation of LRs. Culturing the *35S*::*WUS-GR* plants without Dex leads to the formation and growth of numerous LRs that increased with time. Activation of WUS by Dex leads to inhibition of root formation and root growth and promotes embryo formation, shoots regeneration and green callus formation. Images were taken after 4, 13 and 20 days of culturing; Separated images were merged.
(TIF)

S2 Fig WUS at the presence of auxin induces the formation of somatic embryos, shoots and calli on lateral roots. Eleven day-old *35S*::*WUS-GR* seedlings were transferred to MS medium supplemented with auxin (0.5μM NAA) and 10μM Dex. **On one root we can identify different organs and tissues**; **A**. Whitish callus (on the left), green callus (in the middle) and green callus with undefined structures (right). **B**. Leaves and shoot formation similarly to seedling development. **C**. Somatic embryos formed on lateral root with atypical basal-apical polarity. Occasionally multi-fused embryos with two visible radicles (highlighted) and numerous cotyledons were observed (right). Scale bar: A (right) and C (left) 200 μm all others 500 μm.
(TIF)

S3 Fig In excised roots WUS inhibits the responses to auxin but do not induce shoot or embryo formation. Excised roots of 11-day-old *35S*::*WUS-GR* Arabidopsis seedlings were cultured on auxin and Dex for 11 days. LR formation was suppressed just like in the intact seedlings, but there was no regeneration of shoots or somatic embryos.
(TIF)

S4 Fig WUS induction leads to embryos formation that develops in atypical basal-apical polarity. Scanning electron microscopy views of the WUS induced somatic embryos demonstrate the atypical polarity. On the right leaf primordium develops from the embryo apex. Scale bar: 200 μm.
(TIF)
