# Supplementary figures and images for: Shoot stem cell specification in roots by the WUSCHEL transcription factor
Source: PLoS One. 2017 Apr 26;12(4):e0176093. doi: 10.1371/journal.pone.0176093 (PMC5405954; doi:10.1371/journal.pone.0176093)

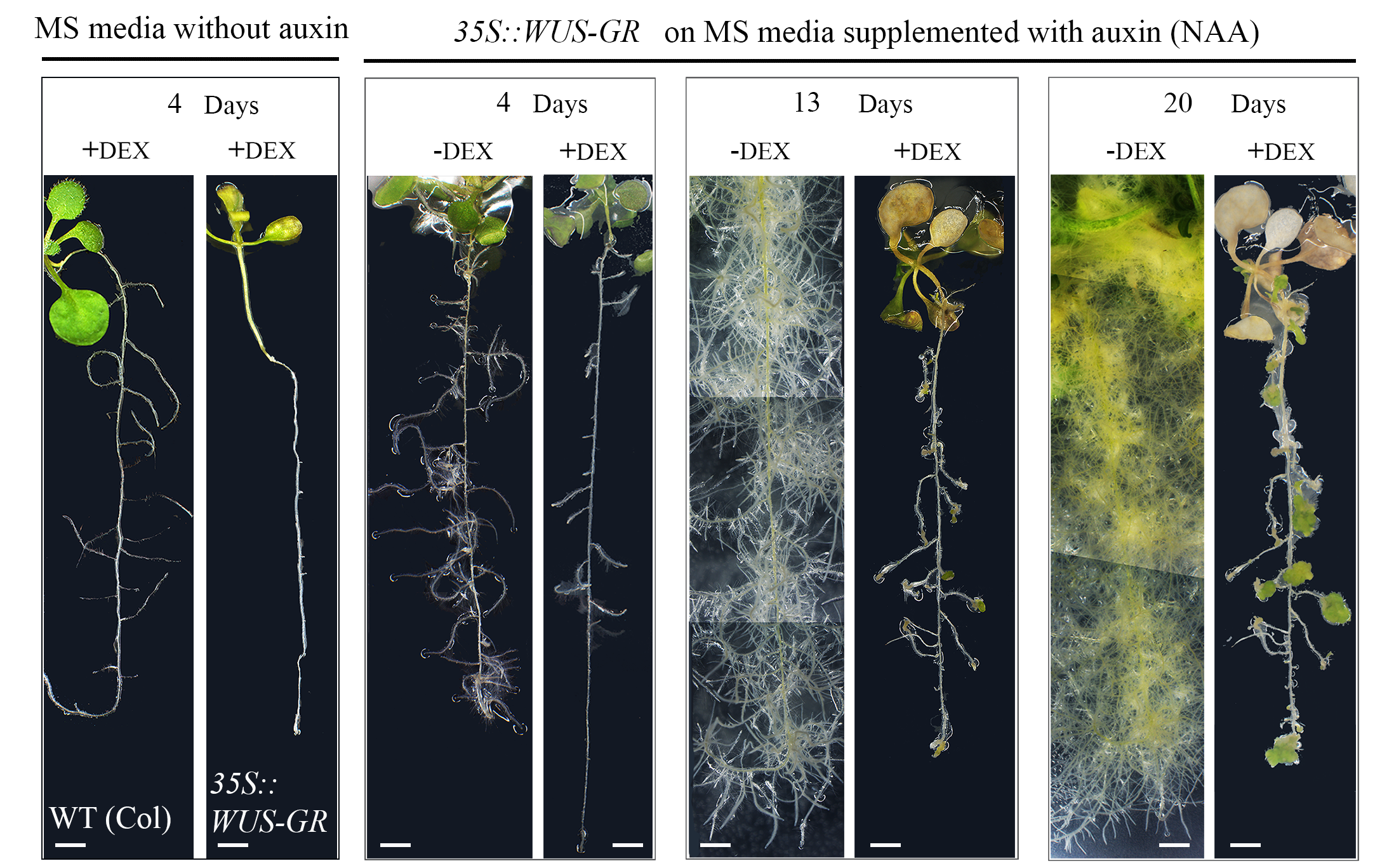

Supplement: S1 Fig — Eleven day-old 35S::WUS-GR seedlings were transferred to medium supplemented with auxin (0.5μM NAA) and 10μM DEX. Left panel: WT Col plants cultured on Dex without auxin show no phenotype; 35S::WUS-GR plants cultured on Dex without auxin exhibit inhibition in root growth and initiation of LRs. Culturing the 35S::WUS-GR plants without Dex leads to the formation and growth of numerous LRs that increased with time. Activation of WUS by Dex leads to inhibition of root formation and root growth and promotes embryo formation, shoots regeneration and green callus formation. Images were taken after 4, 13 and 20 days of culturing; Separated images were merged. (TIF) [file pone.0176093.s003.tif]

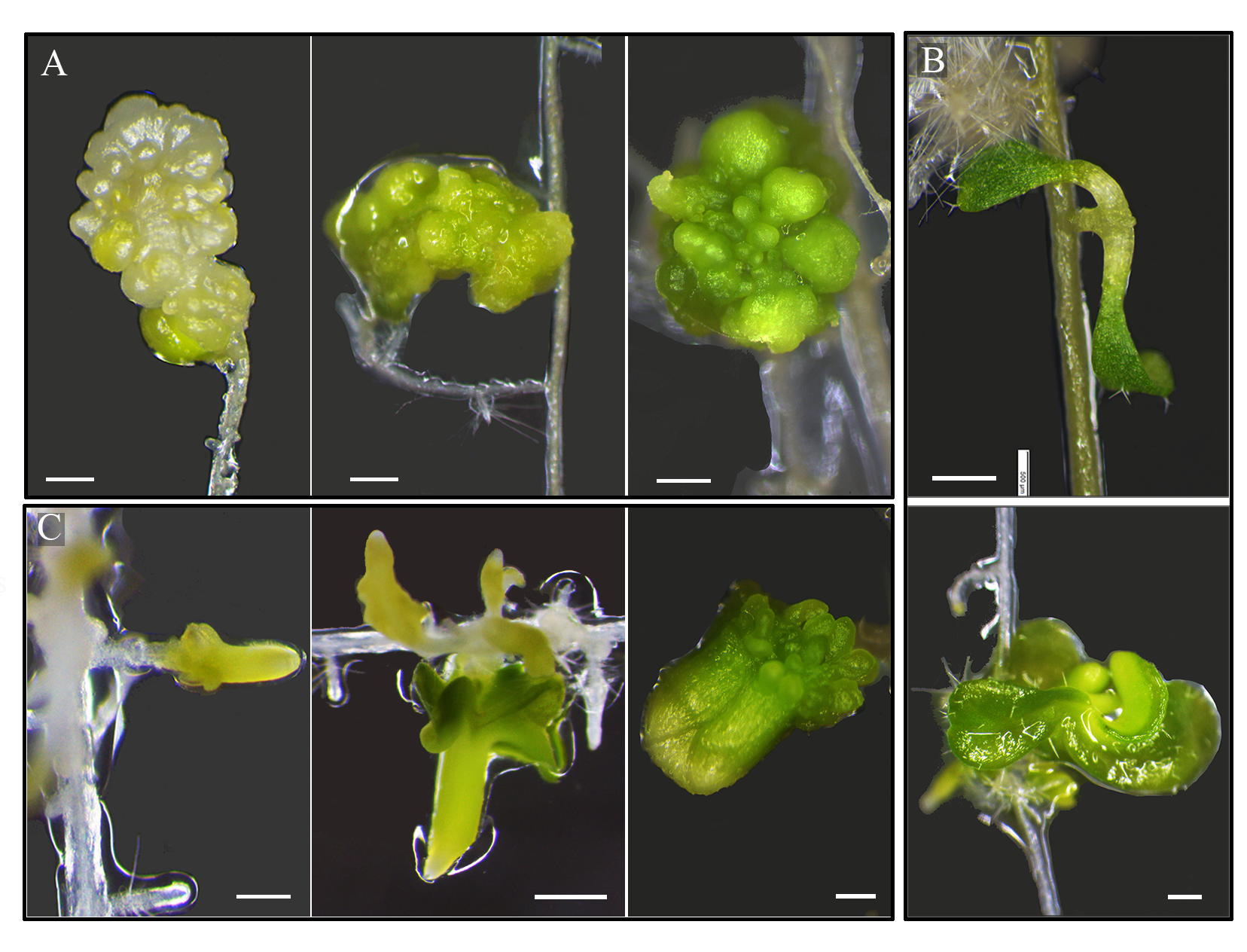

Supplement: S2 Fig — Eleven day-old 35S::WUS-GR seedlings were transferred to MS medium supplemented with auxin (0.5μM NAA) and 10μM Dex. On one root we can identify different organs and tissues; A. Whitish callus (on the left), green callus (in the middle) and green callus with undefined structures (right). B. Leaves and shoot formation similarly to seedling development. C. Somatic embryos formed on lateral root with atypical basal-apical polarity. Occasionally multi-fused embryos with two visible radicles (highlighted) and numerous cotyledons were observed (right). Scale bar: A (right) and C (left) 200 μm all others 500 μm. (TIF) [file pone.0176093.s004.tif]

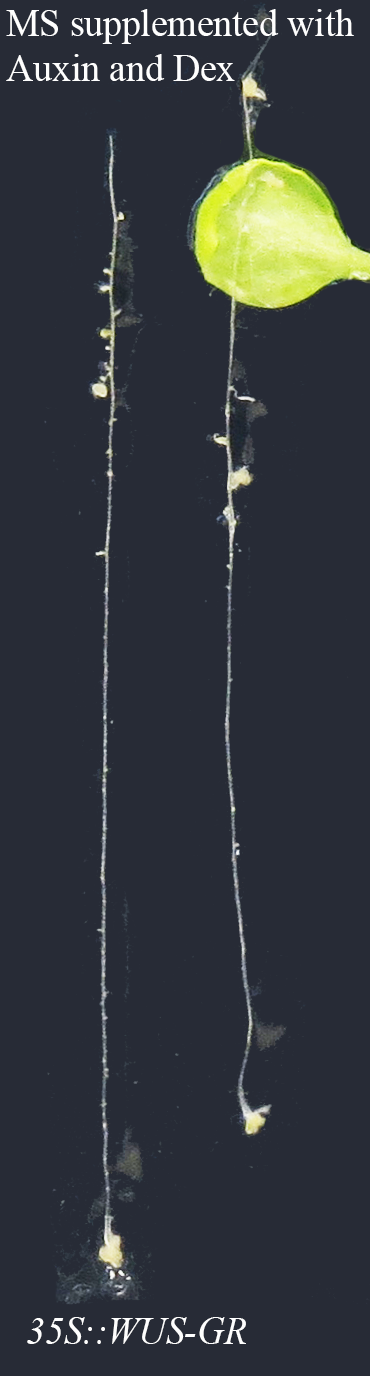

Supplement: S3 Fig — Excised roots of 11-day-old 35S::WUS-GR Arabidopsis seedlings were cultured on auxin and Dex for 11 days. LR formation was suppressed just like in the intact seedlings, but there was no regeneration of shoots or somatic embryos. (TIF) [file pone.0176093.s005.tif]

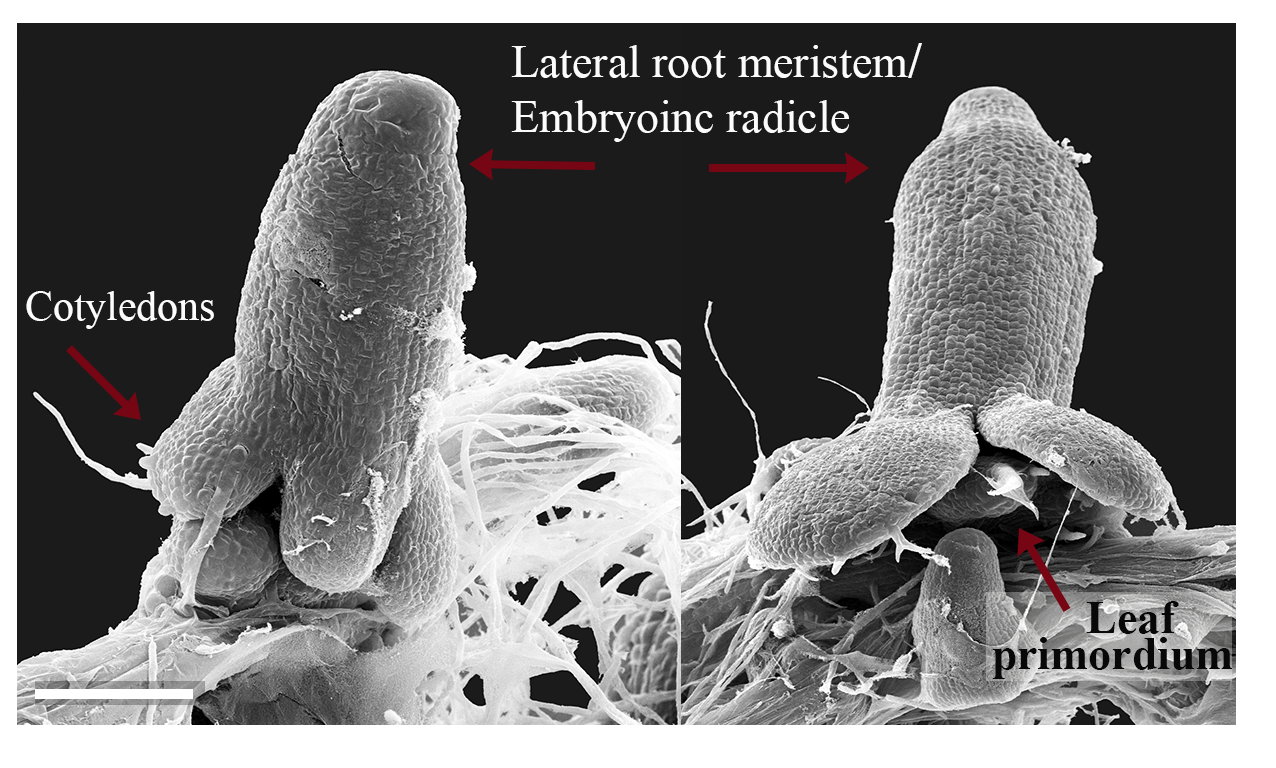

Supplement: S4 Fig — Scanning electron microscopy views of the WUS induced somatic embryos demonstrate the atypical polarity. On the right leaf primordium develops from the embryo apex. Scale bar: 200 μm. (TIF) [file pone.0176093.s006.tif]
